# Antimicrobial Properties of Newly Developed Silver-Enriched Red Onion–Polymer Composites

**DOI:** 10.3390/antibiotics13050441

**Published:** 2024-05-14

**Authors:** Judita Puišo, Jonas Žvirgždas, Algimantas Paškevičius, Shirin Arslonova, Diana Adlienė

**Affiliations:** 1 Department of Physics, Kaunas University of Technology, Studentų Str. 50, LT-51368 Kaunas, Lithuania; 2 Laboratory of Biodeterioration Research, Institute of Botany, Nature Research Centre, Akademijos Str. 2, LT-08412 Vilnius, Lithuania; jonas.zvirgzdas@gamtc.lt (J.Ž.); algimantas.paskevicius@gamtc.lt (A.P.); 3 Tashkent City Branch of Republican Specialized Scientific—Practical Medical Centre of Oncology and Radiology, Boguston Str. 1, Tashkent P.O. Box 100070, Uzbekistan; shirinhon@gmail.com

**Keywords:** red onion, peel, polyvinyl alcohol, gels, films, silver, nanoparticles

## Abstract

Simple low-cost, nontoxic, environmentally friendly plant-extract-based polymer films play an important role in their application in medicine, the food industry, and agriculture. The addition of silver nanoparticles to the composition of these films enhances their antimicrobial capabilities and makes them suitable for the treatment and prevention of infections. In this study, polymer-based gels and films (AgRonPVA) containing silver nanoparticles (AgNPs) were produced at room temperature from fresh red onion peel extract (“Ron”), silver nitrate, and polyvinyl alcohol (PVA). Silver nanoparticles were synthesized directly in a polymer matrix, which was irradiated by UV light. The presence of nanoparticles was approved by analyzing characteristic local surface plasmon resonance peaks occurring in UV-Vis absorbance spectra of irradiated experimental samples. The proof of evidence was supported by the results of XRD and EDX measurements. The diffusion-based method was applied to investigate the antimicrobial activity of several types of microbes located in the environment of the produced samples. Bacteria *Staphylococcus aureus* ATCC 29213, *Acinetobacter baumannii* ATCC BAA 747, and *Pseudomonas aeruginosa* ATCC 15442; yeasts *Candida parapsilosis* CBS 8836 and *Candida albicans* ATCC 90028; and microscopic fungi assays *Aspergillus flavus* BTL G-33 and *Aspergillus fumigatus* BTL G-38 were used in this investigation. The greatest effect was observed on Staphylococcus aureus, Acinetobacter baumannii, and Pseudomonas aeruginosa bacteria, defining these films as potential candidates for antimicrobial applications. The antimicrobial features of the films were less effective against fungi and the weakest against yeasts.

## 1. Introduction

Scientific research on the antibacterial properties of plant materials was started in the second half of the 19th century when Louis Pasteur noted the first antibacterial properties of garlic in 1858 [1]. Since that time, a vast number of different studies have been performed investigating the antimicrobial properties of plants. It was shown [2,3], that one of the oldest cultivated plants Allium cepa L. (Liliaceae) or onion indicates antimicrobial properties and can be used for the treatment of many diseases. Antibacterial and antifungal properties of onion and onion extracts were discussed in several papers [4,5,6,7,8]. It was found that onion peel has 3–5 times higher amounts of isolated phenolic compounds and quercetin and provides approximately 3–5 times higher activity against bacteria than the edible part of the onion [9]. Investigation of antibacterial assays for the isolated compounds containing water extract of onion peel (Allium cepa) has shown that 2-(3,4-dihydroxy phenyl)-4,6-dihydroxy-2-methoxybenzofuran-3-one indicated selective activity against Helicobacter pylori strains, and 3-(quercetin-8-yl)-2,3-epoxy flavanone was effective against MRSA (multidrug-resistant *Staphylococcus aureus*) and *H. pylori* strains [8]. High antimicrobial activity of red onion peel extracts against bacteria *Escherichia coli*, *Pseudomonas fluorescens*, and *Bacillus cereus* and fungi *Aspergillus niger*, *Trichoderma viride*, and *Penicillium cyclopium* was found as well [9]. This indicated the potential of onion peels to be applied in antimicrobial compositions. On the other hand, the utilization of red onion peel as an antimicrobial substance may contribute to the reduction in the problem related to the accumulation of a high amount of waste produced due to the increased market of processed onion [10,11,12,13].

It is known [14] that some metal-based nanoparticles possess antimicrobial activity as well. Among different metallic NPs, silver nanoparticles (AgNPs) play an enormous role as antimicrobial agents [15]. However, there are some issues with the fabrication of AgNPs. The synthesis of AgNPs can be achieved both chemically and physically. Both approaches include drawbacks: physical operations usually fail to regulate particle sizes in the nanoscale range and irregularly sized particles are created. Chemical synthesis of NPs considers the application of toxic materials and requires high energy resources. Despite the indicated drawbacks, the antimicrobial activity of silver nanoparticles is amazing and attracts the huge attention of researchers. Due to this, antimicrobial properties of composites containing silver nanoparticles have been thoroughly investigated during the last decades [16,17,18]. Analyzed research information revealed that there is a possibility to enhance the antimicrobial activity of compounds containing AgNPs, exploring environmentally friendly green synthesis methods for gaining silver nanoparticles [19,20,21]. The green synthesis method has gained a lot of attraction since metallic NPs can be synthesized biologically using various plants and their extracts which are easily available in huge quantities. The plants and their extracts are safe to handle, less toxic, and eco-friendly. Created nanoparticles are biocompatible and efficient against microbial intrusion. It should also be noted that plant extracts act as NP reductants and stabilizers. Several studies discussed the application of onion peel for green synthesis of silver nanoparticles [22,23].

Taking into account that polymer films originally containing AgNPs may be of advantage for their antimicrobial applications, the use of red onion peel extract for green synthesis of silver nanoparticles in polymer films directly was introduced in our previous work [24]. Gelatine (natural polymer) as a matrix for AgNPs has been considered due to its biodegradability, biocompatibility, and unique biological properties. However, gelatine samples exhibited poor mechanical and adhesive properties and were temperature-sensitive, thus limiting their end-use applications [25,26,27,28,29,30,31,32,33,34]. To overcome this problem, PVA, a non-toxic, biodegradable, flexible synthetic polymer possessing characteristics of hydrophilic nature, was suggested as a substitute for gelatine [29,35]. PVA has a good film formation capability, solid conglutination, and excellent thermal stability [36,37,38]. It indicates good forming and manufacturing features and is already adopted for soft tissue replacement (in its hydrogel form) [38]. It is noticed that PVA itself and also gelatin do not possess any antioxidant and antimicrobial properties and do not impact the antimicrobial properties of bioactive materials [39,40,41,42,43,44,45].

Taking into account the results of the conducted literature analysis, our study is aimed at the fabrication and characterization of red onion peel–PVA compositions containing silver nanoparticles and assessment of their antimicrobial efficacy when interacting with different types of microorganisms (bacteria, yeast, fungi).

## 2. Results and Discussion

### 2.1. Optical Properties of Red Onion Peel–PVA Gels Containing Green Synthesized Silver Nanoparticles

**Figure 1:**
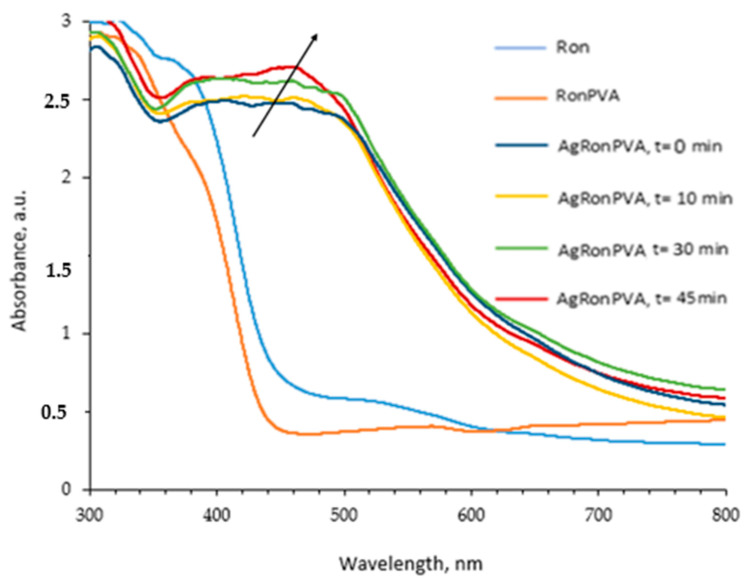
UV-Vis spectra of experimental samples: red onion extract (Ron); RonPVA hydrogel; and AgRonPVA gel after UV exposure for 0 min, 10 min, 30 min, and 45 min.

Red onion extract was prepared following the procedure described in our previous paper [24]. The composition of the prepared samples is indicated in the Materials and Methods chapter. UV-Vis absorbance spectra were used for the identification of the optical properties of different material compositions (Figure 1). Dark-red-colored aqueous red onion peel extract “Ron” indicated a broad low-intensity peak with a maximum of 538 nm. The addition of a small amount of Ron extract to a colorless PVA–water solution resulted in a color change to light pink confirming the formation of a RonPVA hydrogel with an almost negligible UV-Vis peak at 562 nm due to the high dilution of the Ron extract. The concentration of the red onion extract was chosen so that the UV-Vis absorbance peaks corresponding to red onion anthocyanins did not obscure the local surface resonance peaks that indicate the presence of silver nanoparticles in the gels [24]. The addition and dissolving of silver nitrate in the RonPVA hydrogel solution under continuous stirring has not introduced significant color changes, and the formed AgRonPVA gel demonstrated almost the same transparent color, but the observed UV-Vis absorbance peak at 447 nm was shifted towards a lower wavelength (Figure 1).

A possible green-synthesis mechanism was provided by H. Yang et al. [46]. Based on the findings provided in the mentioned article, it was suggested that red onion peel extract can contribute to the green synthesis of nano seeds/nanoparticles and can be used to reduce Ag^+^ and form Ag/Ag_2_O/AgO nanocomposites. It should be noted that the strong oxidation property of nanocomposites can be better addressed by providing an ion-associated form of the composite: Ag/Ag^+^/Ag^3+^. Our suggestion was based on the fact that red onion peel comprises different molecular substances. Proteins and polyphenols and others containing strong reducible hydroxyl groups are among them. The hydroxyl group can reduce silver ions into silver. On the other hand, biological macromolecules containing amino and carbonyl groups exhibit a strong complexation for silver and silver ions, which can be wrapped on the surface of AgNPs providing the effect of dispersion and protection.

The free energy (surface tension) on the surface of nanoparticles, especially when newly formed, is very high, so the nanoparticles are unstable and can easily agglomerate into larger particles. In our case, silver nanoparticles are wrapped by biological macromolecules of red onion peel extract, resulting in reduced surface free energy of the particles and avoiding to some extent particle aggregation and their growth. It should be noticed that in the case of the same volume, the lowest energy of the system is provided when spherical particles are present because spherical-shaped particles occupy the smallest surface area [46].

Based on the information provided above, we assumed that red onion peel extract contributed to the formation of spheric AgNPs as silver composites containing silver (Ag) and silver oxides, Ag_2_O(Ag^+^) and AgO(Ag^3+^), after the addition of AgNO_3_ to the peel extract, as it can be proven by the appearance of the LSPR peak at 447 nm of the UV-Vis spectrum (Figure 1).

However, it should be noted that the influence of the PVA matrix for accommodation of AgNPs is not fully disclosed. Also, the mechanism of dispersion and stabilization of AgNPs coated by biological macromolecules needs further development [47,48,49], especially when speaking about two opposite processes: formation and preservation of AgNP growth by plant extracts and initiation of AgNP growth by photoreduction (UV exposure).

(1) $${Ag}^{+}+\mathrm{Phytochemicals}\;\mathrm{of}\;\mathrm{red}\;\mathrm{onion}\overset{h\nu }{\to }{Ag}^{0}\left(Ag\;NPs\;with\;{Ag}_{x}O_{y}\;coating\right)$$

Polymer hydrogels containing AgNO_3_ were exposed to UV light for 10 min, 30 min, and 45 min. UV-Vis absorbance spectra of differently exposed samples were used to follow up the formation of AgNPs (Figure 1).

It was found that UV-exposed gel samples obtained a reddish-brown color. The appearance of a broad UV-Vis absorbance peak with the maximum intensity at 448 nm was observed after gel exposure for 10 min. The intensity of the peak was slightly increasing with the prolonged duration of UV exposure, and the peak maximum position itself was slightly shifted to a longer wavelength (462 nm) after gel exposure for 45 min. No significant color changes of exposed gels were observed. The observed peaks were identified as local surface plasmon resonance (LSPR) peaks that were characteristic for metal particles introduced in a polymer matrix. It was suggested that the formation of silver nanoparticles (AgNPs) starts from silver seeds which can be created when silver ions bind hydrated electrons and reactive water radicals produced as a consequence of UV exposure or interact with fragments of the red onion since plant extracts are known as reductants and stabilizers for nanoparticles [19]. Created silver seeds grew to silver nanoparticles by binding silver ions on single seeds. The growth of silver nanoparticles is reflected by the broadening of the LSPR peak with a shift towards longer wavelengths. Based on the analysis of the obtained UV-Vis spectra it was assumed that in this experiment, silver nanoparticles of approximately the same size, but in different amounts, were formed depending on the duration of the exposure, rather than growing in size.

**Figure 2:**
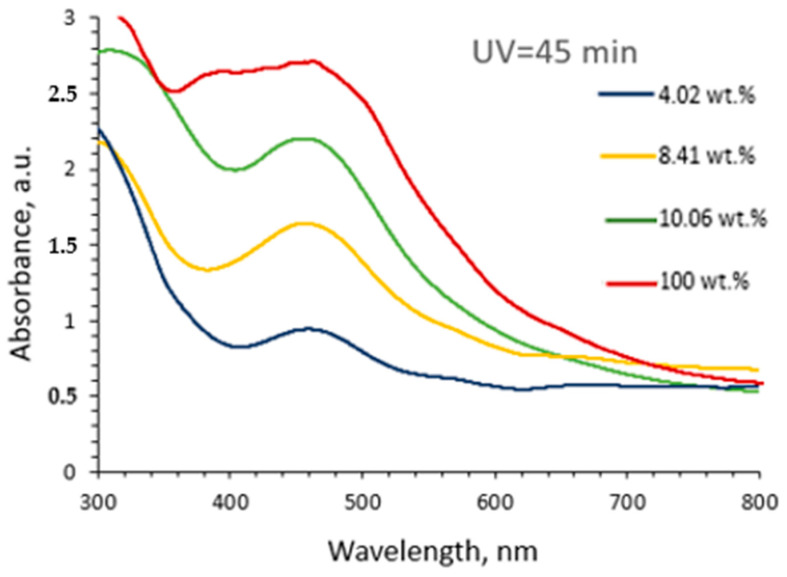
UV-Vis spectra of the diluted in distilled water AgRonPVA gels: 4.02 wt.%; 8.41 wt.%; 10.06 wt.%; 100 wt.% (not diluted AgRonPVA gel).

To identify the influence of formed AgNPs on the optical properties of newly composed hydrogels, different amounts of AgRonPVA gels exposed to UV for 45 min were diluted in distilled water, and the UV-Vis absorbance spectra of these solutions were investigated (Figure 2). It was found that the intensity of the absorption peak was decreasing depending on the amount of AgRonPVA in distilled water. Dilution of the AgRonPVA gel led to the appearance of well-expressed LSPR peaks. The maximum of the LSPR peak of diluted gels remained at the same initial position of (462) nm.

It is known [50,51] that the increasing number of formed silver nanoparticles may change the dielectric properties of gels, causing overlapping of SPR peaks from individual nanoparticles and initiating the shift of LSPR peak to longer wavelengths. Results obtained in this study supported the hypothesis that synthesized spherical AgNPs are accommodated in the cells of the RonPVA polymer networks. Since AgNPs do not aggregate into local metallic silver derivatives in distilled water (dielectric), they can be distributed in the AgRonPVA water solution without the original size change.

### 2.2. Optical Properties of Red Onion Peel–PVA Films Containing Silver Nanoparticles

**Figure 3:**
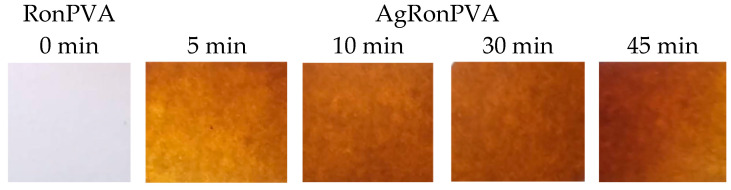
Photographs of fabricated experimental films.

Antibacterial films containing AgNPs can be fabricated following preset requirements: (1) the avoidance of AgNP agglomeration and formation of metallic silver; (2) preservation of antimicrobial properties of the AgRonPVA gels during the film formation procedure. Also, ~10% loss in mass during the drying process of gels (formation of films) was taken into account. Photographs of the UV-exposed AgRonPVA films are provided in Figure 3 alongside a photograph of the colorless RonPVA film (without AgNPs) which is shown for comparison.

**Figure 4:**
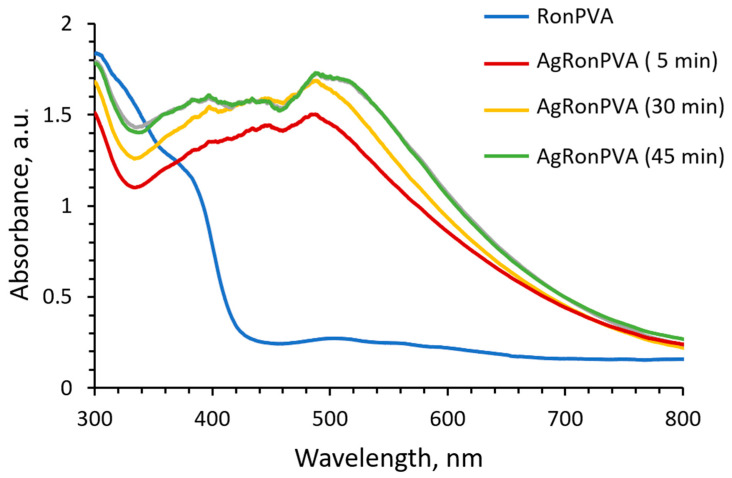
UV-Vis spectra AgRonPVA films obtained from 100 wt.% AgRonPVA gels exposed to UV.

Due to the loss of some water amount by drying of exposed gels, the UV-Vis spectra of films (Figure 4) indicated broadening of the LSPR peaks that were better pronounced and slightly shifted towards longer wavelength as compared to those of the gels shown in (Figure 1). LSPR peak locations of the differently exposed AgRonPVA films were found by eliminating baseline and fitting: locations of LSPR peak maximum at 486 nm and 492 nm were found for 5 min UV exposed film and 45 min exposed film, respectively. A weak LSPR peak with a maximum of 507 nm was found for the RonPVA films. It was slightly shifted towards a lower wavelength as compared to the RonPVA gels indicating the contribution of anthocyanins from red onion peel extract that appeared due to the loss of water during the drying process and were responsible for color changes of the films [24,52]. Taking into account the appearance of only one single LSPR peak and its location in the UV-Vis spectra, it is assumed that only spherical or near-spherical particles were synthesized. In the case of anisotropic particles, two or three LSPR peaks would be seen by their shapes [53]. As the AgRonPVA gel dries, water evaporates from the samples, changing the environment of the silver nanoparticles to an optically denser one (polymer network), limiting particles’ mobility in the volume and reducing agglomeration possibility. This results in an increase in the SPR peak intensity as compared to the LSPR peak measured in the gels (Figure 2).

**Figure 5:**
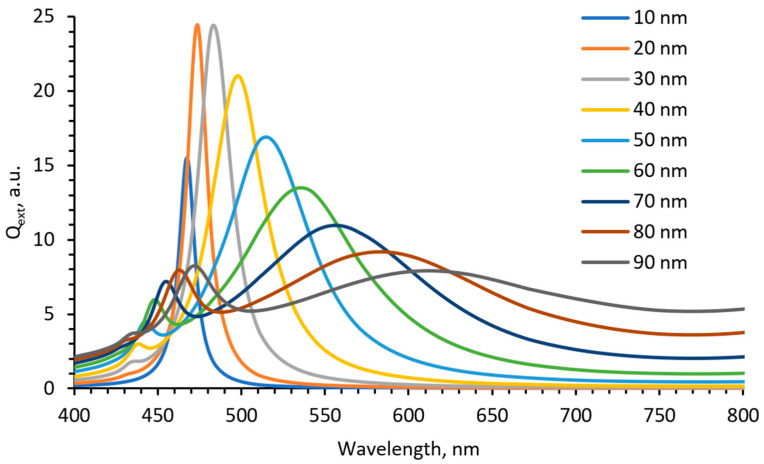
Simulated UV-Vis spectra for differently sized spherical silver nanoparticles in PVA.

It is assumed that during drying of the film, the water amount in gels will be reduced, which leads to the changes in the polymer matrix. Since the matrix network becomes denser, the growth in the AgNPs from the seeds is restricted. Possible growth and aggregation of silver nanoparticles were simulated using open access “MiePlot” software, version 4.6 [54]. The algorithm of this software is based on calculations of Mie scattering from a sphere (scattering of light by Ag NPs in our case). “MiePlot” software, version 4.6 allows us to calculate scattering efficiency (Q_ext_—extinction; Q_abs_—absorption; Q_s_—scattering) as a function of wavelength. Light absorption dominates in the extinction (extinction = absorption + scattering) spectrum for particles with a relatively small (radius <20 nm), and light scattering becomes the dominant process for larger particles. In our calculations, we used PVA as a medium for the dispersion of nanoparticles having a diameter from 10 to 90 nm with a 5% standard deviation in a log-normal distribution. Q_ext_ as a function of wavelength is presented in Figure 5.

The performed simulation revealed that only small particles (<40 nm) are indicated by strong well-defined peaks. Increasing the particle size leads to a decrease in the SPR peak intensity, peak broadening, and shift towards a longer wavelength. A comparison of simulation and experimental results leads to the conclusion that >40 nm sized AgNPs were mainly formed in our experiment. However, performed modeling has not accounted for the water content in the films and also the presence of the red onion peel extract, which may affect the SPR peak position.

**Figure 6:**
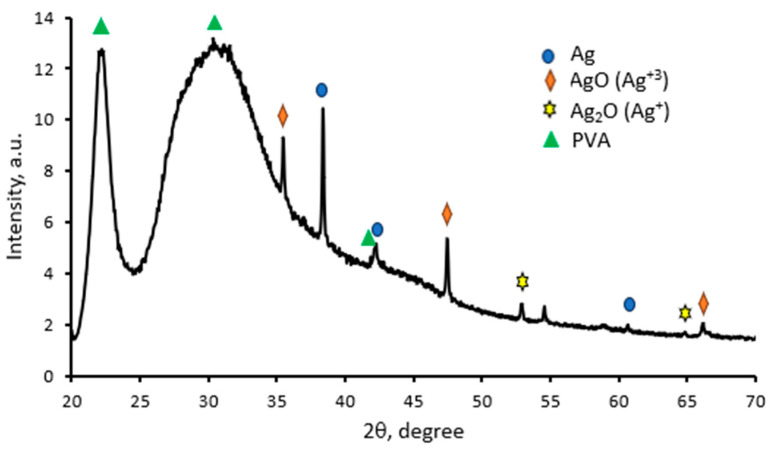
XRD pattern red onion PVA films containing silver nanoparticles (AgRonPVA45).

It is known that the crystalline structure of polymer composites can be estimated from the XRD patterns. The XRD pattern of the 45 min UV-exposed AgRonPVA film is presented in Figure 6.

The XRD peaks corresponding to silver nanoparticles located in the PVA matrix are of a lower intensity in comparison with AgNPs produced in gels, because Ag nanoparticles are integrated into the PVA network [55]. Silver nanocrystals are usually characterized by a face-centered cubic (fcc) structure with a dominating orientation of crystallites along the (111) direction (38.45°). The XRD peaks of lower intensity are seen at 42.30° and 60.70° corresponding to (200) and (220) crystalline planes of the face-centered cubic crystalline structure of silver [JCPDS No. 04-0783, JCPDS file no. 84-0713]. An additional broad peak at 30.6° and distinct peaks at 22° and 41.8° were assigned to corresponding PVA planes (110) and (200), as was indicated in [55,56,57,58,59]. A Ag crystallite size of 53.96 nm in AgRonPVA films was determined using the Scherrer method [60,61,62].

H.Yang et al. [46] indicated that silver derivatives, Ag, ${Ag}_{2}O,$ and AgO, may be registered, suggesting their green synthesis in the bio-organic phase and crystallization on the surface of Ag nanoparticles. This explains the presence of the additional diffraction peaks observed in our XRD pattern (Figure 6): AgO(${Ag}^{3+}$) (JCPDS, file No. 84-1108) and ${Ag}_{2}O$(${Ag}^{+}$) (JCPDS, file No. 42-0874).

**Figure 7:**
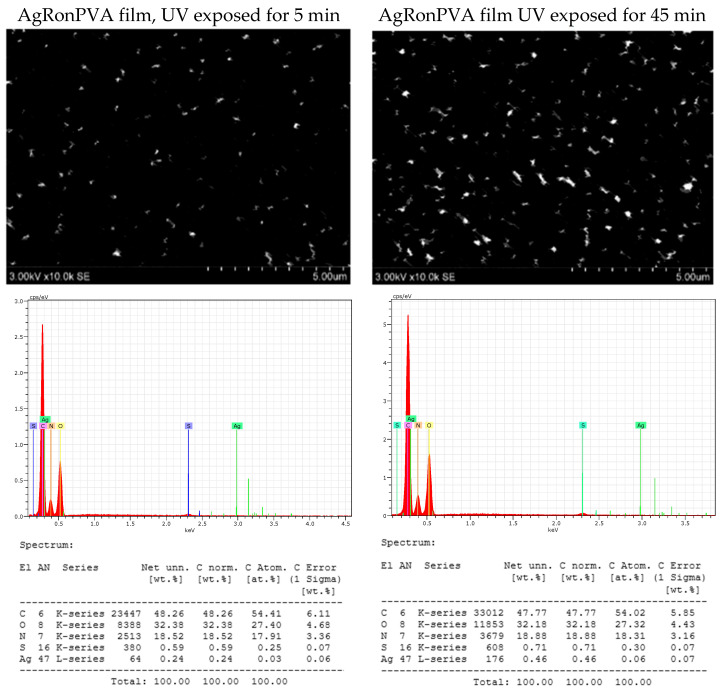
SEM images and EDX analysis results.

Analysis of SEM images and results of EDX evaluation of unexposed (as prepared) and UV-exposed AgRonPVA films revealed that the observation of AgNPs was very problematic, due to the low amount of seeds/particles in the film and their embedding in the network of the polymer matrix. Due to the very small amount of silver, it was not detected in as-prepared AgRonPVA films. An example of the obtained results of differently UV-exposed AgRonPVA films is provided in Figure 7.

It was found that the amount of silver in as-prepared and 5 min UV-exposed films was low—0.24 wt.%—indicating the possible contribution of red onion peel extract to green synthesis of nano seeds. The registered amount was slightly increasing with the increased duration of UV exposure and was 0.29 wt.% after film UV exposure for 10 min, 0.39 wt.% after 30 min, and 0.46 wt.% after 45 min.

### 2.3. Antimicrobial Activity of the AgRonPVA Films Containing Silver Nanoparticles

Bacteria *Staphylococcus aureus* ATCC 29213, *Acinetobacter baumannii* ATCC BAA 747, and *Pseudomonas aeruginosa* ATCC 15442; yeasts *Candida parapsilosis* CBS 8836 and *Candida albicans* ATCC 90028; and microscopic fungi *Aspergillus flavus* BTL G-33 and *Aspergillus fumigatus* BTL G-38 were used for the assessment of the antimicrobial activity of experimental AgRonPVA films. The agar diffusion method was used for antimicrobial activity testing of red onion skin extract films containing silver nanoparticles. This was realized by the uniform spreading of the prepared microorganism suspensions (see Section 3) with a swab on Mueller–Hinton agar (bacteria) or on Sabouraud dextrose agar (yeasts and microscopic fungi) in Petri dishes. The pieces of AgRonPVA films (5 mm × 5 mm) containing silver nanoparticles were placed on the surface of each media as the microbial suspension was absorbed into it.

It is to point out that red onion peel extract itself (the main bioactive agents are phenolic compounds) or in combination with silver nanoparticles provides inhibition properties against different bacteria as was shown by several authors: *Streptococcus agalactiae* (Gram-positive), *Pseudomonas aeruginosa* (Gram-negative), *Salmonella typhimurium* (Gram-negative), and *Staphylococcus aureus* (Gram-positive) [63]. The antimicrobial compounds made of red onion peel have various mechanisms to inhibit bacteria: inhibition of phagocytosis in macrophages; increasing the production of Interleukin-12 (IL-12) by quercetin and thus increasing the phagocytic ability of macrophages; altering the membrane potential of bacterial cells and disrupting their performance; and binding to bacteria pili and inhibiting bacterial adhesion [64]. The red onion skin extract also indicates antifungal properties against some fungi, however to a lesser extent and with lower effectiveness as compared with bacteria [9,65,66].

Three antibacterial mechanisms are known when discussing the antimicrobial properties of silver nanoparticles [18,67,68]; however, it should be noted that the chemical size, charge, and surface structure of silver nanoparticles influence their antibacterial capacity as well [69].

The first mechanism proposes that silver nanoparticles might penetrate the outer membrane accumulating in the inner membrane, where the adhesion of the nanoparticles to the cell generates their destabilization and damage, increasing membrane permeability and inducing leakage of cellular content and subsequently its death [70].

The second mechanism proposes that nanoparticles not only can break and cross the cell membrane altering its structure and permeability but can also enter the cell where silver nanoparticles may interact with sulfur or phosphorus groups, present in intracellular content such as DNA and proteins, altering their structure and functions [70]. Silver nanoparticles also may alter the respiratory chain in the inner membrane by interacting with thiol groups in the enzymes inducing reactive oxygen species and free radicals, generating damage to intracellular machinery and activating the apoptosis pathway.

The third mechanism may be present in parallel with two others and accounts for the release of silver ions from the nanoparticles, which due to their size and charge can interact with cellular components altering metabolic pathways, membranes, and even genetic material [71,72,73,74,75,76].

Based on the results obtained investigating experimental AgRonPVA films containing composite-type silver NPS, it was assumed that the third mechanism of the antimicrobial activity was responsible for the inhibition of different microorganisms.

**Figure 8:**
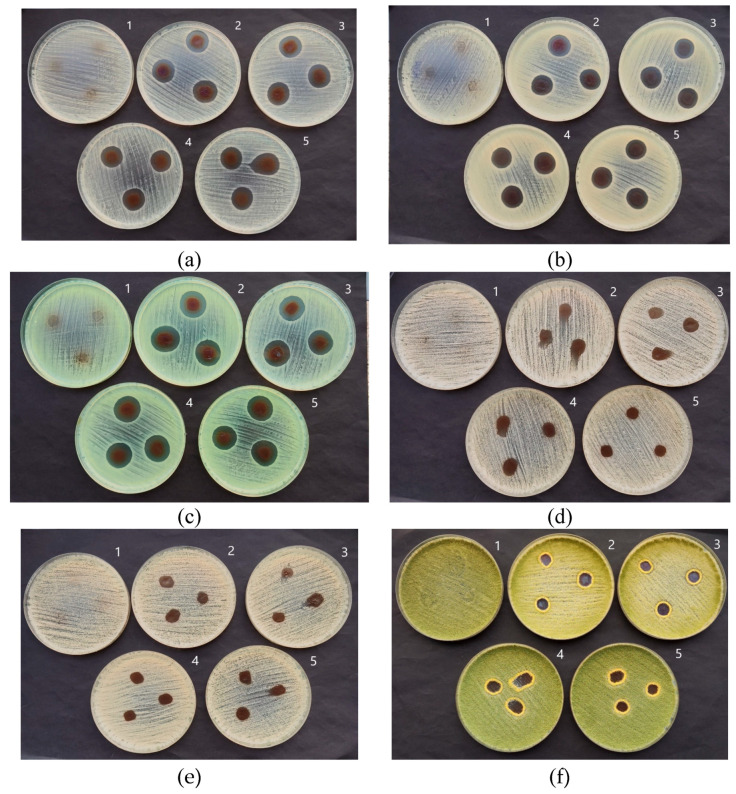
Antimicrobial activity of AgRonPVA films containing silver nanoparticles against (**a**) *Staphylococcus aureus* ATCC 29213, (**b**) *Acinetobacter baumannii* ATCC BAA 747, (**c**) *Pseudomonas aeruginosa* ATCC 15442, (**d**) *Candida parapsilosis* CBS 8836, (**e**) *Candida albicans* ATCC 90028, and (**f**) *Aspergillus flavus* BTL G-33 fungus. Numbering of Petri dishes with immersed films: (1) control; (2) AgRonPVA (UV 5 min); (3) AgRonPVA (UV 10 min); (4) AgRonPVA (UV 30 min); (5) AgRonPVA (UV 45 min).

The results of the performed study have shown (Table 1 and Figure 8) that all differently exposed (duration of exposure was responsible for the formation of a certain number of AgNPs) AgRonPVA films indicated antimicrobial activity against applied bacteria, yeasts, and fungi. The greatest effect was observed on *Staphylococcus aureus*, *Acinetobacter baumannii*, and *Pseudomonas aeruginosa* bacteria. The diameter of the inhibition zones of the tested bacteria ranged from 20.0 mm (UV 5 min) to 18.7 mm (UV 45 min) for *Staphylococcus aureus*, from 18.3 mm (UV 5 min) to 18.0 mm (UV 45 min) for *Acinetobacter baumannii*, and from 21.0 mm (UV 5 min) to 19.7 mm (UV 45 min) for *Pseudomonas aeruginosa* and indicated decreasing tendency with the increasing duration of UV exposure. The antimicrobial features of the AgRonPVA films were less effective against fungi, where the diameter of inhibited zones varied between 13.0 mm (UV 5 min) and 12.6 mm (UV 45 min) for *Aspergillus flavus*, and between 16.0 mm (UV 5 min) and 11.7 mm (UV 45 min) for *Aspergillus fumigatus* which indicated the highest sensitivity to the film exposure duration. The antimicrobial effect of the films on yeasts (*Candida parapsilosis* and *Candida albicans*) was the weakest.

Obtained results are in line with the findings of Pereira et al. [77], who showed that a combination of plant extracts, possessing antimicrobial properties and polymers containing AgNPs, leads to an enhanced antimicrobial effect compared with these compounds alone, thus indicating conjugates as promising candidates for biocidal treatments.

## 3. Materials and Methods

### 3.1. Preparation of AgRonPVA Gel

Fresh bulbs of red onions (Allium cepa ‘Red Karmen’) were purchased from a local vegetable market. Onion peel extract was prepared following instructions provided in our previous work [24]. Particularly, peels of fresh red onion were removed from the bulbs and dried. A total of 2.65 g of dried red onion peel was immersed in 53 g of distilled water and heated for 20 min at 62.5 °C. The prepared extract was filtered twice using a white-band filter (Filtrak, Germany, size of pores 8–12 µm) and was ready to use.

AgNO_3_ (CAS No 7761-88-8, purity ≥99.9%, Sigma–Aldrich, Poznan, Poland) was in distilled water to achieve a 1 molar (M) concentration of silver nitrate solution.

PVA (C_2_H_4_O)_n_ powder (CAS 25213-24-5, Chempur^®^, Merck KGaA, Darmstadt, Germany). All was dissolved in distilled water to achieve 10% concentration under continuous stirring keeping a constant temperature of 60 °C. Later, the PVA solution was cooled down to room temperature.

### 3.2. Synthesis of AgNPs in RonPVA Gels

In total, 200 μL of 1 M AgNO_3_ water-based solution was dripped into 4 g of freshly prepared “Ron” extract and filled with 76 g of 10 wt.% of PVA water-based solution under continuous stirring. In total, 20 g of the AgRonPVA gel was poured into glass bottle (22 ± 0.5 m, LBG SVSN-C26-121), and the samples were ready for investigation. The composition of experimental samples is provided in Table 2.

Photoreduction of silver ions in the solution was achieved by exposing experimental samples to a UV lamp (36 W black light source with a UV emission peak at 365 nm and weak blue light emission peak at 404 nm). The exposure time varied from 5 to 45 min. Exposed AgRonPVA gels were stored in the dark for at least 24 h.

### 3.3. Preparation of AgRonPVA Films

AgRonPVA films were formed via drying of a small amount (5 g) of the UV-exposed pure (100 wt.%) AgRonPVA gel deposited as a thin layer in a Petri dish. To avoid thermal and photo effects that may contribute to the loss of antimicrobial properties and initiate the growth and aggregation of silver nanoparticles, Petri dishes (55 mm) with the deposited gels were dried in the dark box at room temperature (20 ± 2 °C) for 24 h.

The thickness of the dried films prepared from 5 g of gels was (156 ± 15) µm. The average loss in mass of AgRonPVA films related to the drying process was (10.1 ± 0.3) wt.%.

### 3.4. Characterization of the AgRonPVA Gels and Films

Optical properties (absorbance) of the UV exposed samples were evaluated by analyzing UV-ViS absorption spectra of experimental gels and films obtained using Photospectrometer Ocean Optics (measurement range from 200 to 1100 UV/Vis, bandwidth 1.5 nm Ocean Optics, Ocean Optics, Inc., Orlando, FL, USA).

The thickness of the films was obtained by measuring with a digital micrometer 0-25MM IP65 (measurement range 0–25 mm, resolution ±0.002 mm, accuracy: ± 4µm (HEBDA Werksvertretungen E.K., Freiburg im Breisgau, Germany).

The structure of synthesized Ag nanoparticles was investigated using a D8 Discover X-ray diffractometer (Bruker AXS GmbH, Karlsruhe, Germany) operating at 40 kV and 40 mA with a Cu Kα radiation source (λ = 1.5418 Å) and parallel beam geometry with 60 mm Göbel mirror. Diffraction patterns were recorded using a fast-counting LynxEye detector with an opening angle of 2.475° and a slit opening of 6 mm. The peak intensities were scanned over the range of 20–7° (coupled 2θ–θ scans) using a step size of 0.05° and a collection time of 60 s per step.

The surface morphology of red-onion-PVA films without and with silver nanoparticles were investigated by a scanning electron microscope (SEM, Hitachi S-3400 N, Tokyo, Japan) using a secondary electron detector.

Elemental mapping of red onion PVA films without and with silver nanoparticles was performed using energy-dispersive X-ray spectroscopy (EDS, Bruker Quad 5040, Hamburg, Germany).

### 3.5. Microorganisms and Inoculum Preparation

Bacteria *Staphylococcus aureus* ATCC 29213, *Acinetobacter baumannii* ATCC BAA 747, and *Pseudomonas aeruginosa* ATCC 15442, yeasts *Candida parapsilosis* CBS 8836 and *Candida albicans* ATCC 90028, and microscopic fungi *Aspergillus flavus* BTL G-33 and Aspergillus fumigatus BTL G-38 were used in assays. Microorganisms were stored at −70 °C in a freezer in the Laboratory of Biodeterioration Research of the Nature Research Centre (Vilnius, Lithuania). Bacteria for antimicrobial tests were grown on Tryptone Soy Agar (Merck, KGaA, Darmstadt, Germany), and yeasts and fungi were grown on Sabouraud dextrose agar (Merck, KGaA, Darmstadt, Germany). Inoculums were obtained from overnight bacterial cultures grown at 37 ± 1 °C. Yeasts were cultured for 2 days, and fungi were cultured for 4 days at 28 ± 1 °C. The optical density of the microorganism cell suspensions was measured with a spectrophotometer (Thermo Scientific, Waltham, MA, USA) at 530 nm for yeasts and microscopic fungi and at 625 nm for bacteria. The resulting microorganism suspensions were vortexed for 15 s.

### 3.6. Evaluation of the Antimicrobial Activity of the AgRonPVA Films Containing Silver Nanoparticles

The agar diffusion method was used for antimicrobial activity testing of AgRonPVA films containing silver nanoparticles. For the agar diffusion assay, each microorganism suspension was uniformly spread with a swab on Mueller–Hinton agar (bacteria) or on Sabouraud dextrose agar (yeasts and microscopic fungi) in 90 mm Petri dishes. The pieces of AgRonPVA films (5 mm × 5 mm) containing silver nanoparticles were placed on the surface of each media as the microbial suspension was absorbed into it. The plates were incubated at 28 ± 1 °C for 2 days (yeasts) or 4 days (microscopic fungi), while the plates with bacteria were incubated at 37 ± 1 °C for 1 day only. All tests were triplicated for all strains. The pure RonPVA film was used as a negative control. The inhibition zone’s diameters were measured in millimeters after the incubation period. Data were collected and processed using open access program packages. Mean values, standard errors, and confidence intervals were estimated.

## 4. Conclusions

AgRonPVA nanocomposites containing silver nanoparticles have been produced at room temperature in the form of gels and thin films. The formation of silver nanoparticles in these composites was achieved by the photoreduction method via exposing experimental samples to UV light and was verified by detecting characteristic SPR peaks in the UV-Vis absorbance spectra. It was shown that green synthesis of silver seeds/nano particles was possible, and red onion peel extract contributed to the final reduction in AgNPs. Mainly spheric AgNPs as silver composites containing silver (Ag) and silver oxides, Ag_2_O(Ag^+^) and AgO(Ag^3+^), were formed.

The antimicrobial activity of the UV exposed AgRonPVA films was investigated using the agar diffusion method. The greatest effect was observed on *Staphylococcus aureus*, *Acinetobacter baumannii*, and *Pseudomonas aeruginosa* bacteria. The antimicrobial features of the films were less effective against fungi and the weakest against yeasts. In general, AgRonPVA films containing silver nanoparticles indicated higher antifungal activity as compared to the previously indicated antimicrobial activity of silver NP-enriched red onion extract–gelatine films [24], thus defining these films as potential candidates for antimicrobial applications.

## Figures and Tables

**Table 1:** Antimicrobial activity of the AgRonPVA films containing silver nanoparticles.

Microorganism	Films				
	1	2	3	4	5
	RonPVA (Control)	AgRonPVA (UV 5 min)	AgRonPVA (UV 10 min)	AgRonPVA (UV 30 min)	AgRonPVA (UV 45 min)
	Zone Diameter, mm				
*Staphylococcus aureus* ATCC 29213	0	20.0 ± 1.0	19.7 ± 0.6	18.7 ± 0.6	18.7 ± 0.6
*Acinetobacter baumannii* BAA 747	0	18.3 ± 0.6	18.0 ± 0.0	18.0 ± 0.0	18.0 ± 0.0
*Pseudomonas aeruginosa* ATCC 15442	0	21.0 ± 0.0	20.7 ± 0.6	20.7 ± 0.6	19.7 ± 0.6
*Candida parapsilosis* CBS 8836	0	10.7 ± 0.4	11.3 ± 0.5	9.3 ± 0.4	9.5 ± 0.3
*Candida albicans* ATCC 90028	0	9.7 ± 0.4	9.3 ± 0.3	9.7 ± 0.4	9.3 ± 0.4
*Aspergillus flavus* BTL G-33	0	13.0 ± 0.5	12.7 ± 0.6	12.0 ± 0.0	12.6 ± 0.4
*Aspergillus fumigatus* BTL G-38	0	16.0 ± 0.0	13.3 ± 0.6	14.3 ± 0.6	11.7 ± 0.5

**Table 2:** Composition of the experimental samples.

Solutions	AgRonPVA (Gel)
Red onion extract, wt.%	4.99
PVA gel wt.%	94.73
1 M AgNO_3_, wt.%	0.28
Total, wt.%	100.00

## Data Availability

Data are contained with the article.
